# Leaf Functional Trait Responses of Urban Street Trees to Point-Source Heat Stress: A Shift Toward Resource-Conservative Strategies Driven by Air-Conditioner Exhausts

**DOI:** 10.3390/plants15131952

**Published:** 2026-06-25

**Authors:** Jiyou Zhu, Hongyuan Li

**Affiliations:** College of Environmental Science and Engineering, Nankai University, Tianjin 300350, China; eialee@nankai.edu.cn

**Keywords:** point-source heat stress, leaf functional traits, leaf economics spectrum, stomatal strategy, resource-conservative strategy

## Abstract

Urban green infrastructure is increasingly exposed to fine-scale thermal heterogeneity generated by anthropogenic point-source heat emissions, yet the leaf-level responses of adjacent vegetation to such localized stress remain poorly understood. Here, we examined whether air-conditioner (AC) exhaust, a widespread point-source heat emitter, is associated with functional trait shifts in *Fraxinus chinensis* street trees, and whether easily measurable leaf traits can serve as candidate indicators for ecological monitoring. Using a matched treatment–control field comparison, we compared trees located 2 m from operating AC units with unaffected controls and quantified nine leaf functional traits together with concurrent microclimate variables. AC exhaust created a distinct compound heat–drought–wind micro-environment at the 2 m patch scale, with higher air temperature (+6.3 °C), lower relative humidity (−12.3 percentage points), and higher wind speed (5.2-fold). Exposed trees showed a coordinated shift toward more resource-conservative leaf traits: leaf dry matter content (+14.8%), tissue density (+13.6%), leaf thickness (+6.3%), and stomatal density (+11.7%) increased significantly, whereas specific leaf area (−10.6%), leaf area (−12.5%), chlorophyll content index (−4.6%), and stomatal area (−10.4%) decreased significantly. The observed “small-and-numerous” stomatal configuration suggests altered stomatal regulation, although its implications for transpiration-driven cooling require direct physiological validation. Exploratory structural equation modeling suggested associations among AC-exhaust exposure, leaf economic strategy, and stomatal traits; stomatal regulation showed the highest proportion of model-explained variance (R^2^ = 0.598), but this value should not be interpreted as direct evidence of impairment severity or restoration potential. Leaf dry matter content, specific leaf area, and stomatal density emerged as sensitive and practical candidate indicators of AC-exhaust-associated leaf functional shifts. These findings support precautionary management near AC exhaust outlets, while specific planting-distance thresholds and zoning frameworks require future validation through distance-gradient or manipulative experiments.

## 1. Introduction

Urban green infrastructure includes street trees, parks, and vegetated corridors. It provides key ecosystem services, including microclimate regulation, carbon storage, and stormwater control [1,2]. These functions may be affected by fine-scale thermal heterogeneity from human heat sources. The urban heat island (UHI) effect is well documented as a city-scale thermal stressor [3]. Yet much less is known about small thermal patches from discrete point sources. These patches may affect nearby plants and create a need for local management. AC exhaust differs from the broader UHI background. It is local, directional, and abrupt. It can change air conditions within a few meters of the outlet. These short-distance thermal anomalies can create steep environmental gradients. Such gradients may reduce plant physiological performance. If widespread, they may also weaken regulating services from urban vegetation. AC exhaust also differs from UHI stress in its stress structure. It combines high air temperature, low relative humidity, and forced airflow. Together, these factors form a compound heat–drought–wind micro-environment. Previous studies show that high vapor pressure deficit can limit stomatal conductance. It can also alter transpiration and increase hydraulic risk. Airflow can further change leaf boundary-layer conductance and gas exchange [4,5]. Although AC exhaust may occur intermittently, it often repeats during the warm season. This repeated exposure may create a chronic disturbance microsite for nearby plants. For this reason, the ecological relevance of AC-exhaust patches should be tested with field evidence. It should not be treated as an established assumption. Trait-based measurements provide a practical way to test this hypothesis.

The leaf economics spectrum (LES) links leaf traits with plant resource-use strategies and ecosystem functions [6,7]. It is useful for assessing plant functional status and informing restoration planning. In urban thermal environments, several traits are especially important. These include specific leaf area (SLA), leaf dry matter content (LDMC), leaf tissue density (LTD), chlorophyll content, and stomatal traits [8]. These traits show how trees balance resource acquisition, stress tolerance, and cooling-related functions. High temperature and increased atmospheric demand can favor more conservative strategies in urban trees [9,10,11,12]. These shifts are often reflected by lower SLA and higher LDMC and LTD. Such trait changes may affect photosynthesis, transpiration, and cooling services. Thus, leaf traits can help link individual plant responses with green-infrastructure function. Most existing studies have focused on macro-scale urban–rural or UHI gradients. They often treat the thermal environment as spatially uniform. Their spatial grain is usually hundreds of meters to kilometers. This scale may miss the fine thermal heterogeneity experienced by individual plants [13,14]. This mismatch limits our ability to detect functional changes caused by small but intense heat sources. It also limits spatially precise planting and restoration design.

Air-conditioner (AC) outdoor units are common point-source heat emitters. Yet their ecological effects are still often overlooked. A typical residential unit releases air 5–15 °C warmer than ambient air. It also has lower humidity and outlet wind speeds of 3.0–4.5 m s^−1^ [15,16]. This discharge can form a small but intense thermal patch. Such a patch may persist for several hours each day during the warm season. It can be described as a “point-source heat landscape.” Building-energy and urban-canopy studies show that AC waste heat can affect outdoor temperature, heat storage, and thermal feedbacks [17,18,19]. These effects have been studied at street, neighborhood, and city scales. However, most studies have focused on energy balance and outdoor thermal comfort. The effects of localized AC exhaust on nearby vegetation remain poorly understood. From a restoration perspective, each AC unit may create a disturbed micro-site. Plants near the outlet may experience repeated heat, dryness, and airflow. These conditions may shift plant traits away from a desired functional state. Although AC units are widespread, their effects on adjacent vegetation remain poorly quantified. Horticultural observations have reported leaf chlorosis and growth inhibition within about 3 m of AC outlets [20]. Yet these observations lack quantitative trait-based evidence. Such evidence is needed to link thermal patch structure with plant functional responses. It is also needed to support planting and management decisions. Current engineering standards, such as GB/T 17790-2025 [15], mainly consider ventilation requirements. Therefore, this study does not assume that planting-distance standards should be revised immediately. Instead, it asks whether ecological indicators can provide complementary evidence for planting design near AC outdoor units.

Here, we investigated whether AC exhausts act as ecologically significant point-source stressors that degrade plant functional performance by reshaping leaf functional traits in *Fraxinus chinensis* Roxb., a widely planted street tree in Beijing. Using a matched treatment–control field comparison with trees located 2 m from operating AC units and control trees outside the thermal influence zone, we measured nine leaf functional traits covering structural, biochemical, LES, and stomatal dimensions, together with microclimatic conditions. We specifically asked: (1) How does this point-source heat stress alter individual leaf traits and the multivariate trait syndrome, and what does this imply for the functional integrity of the affected vegetation? (2) Which traits are most responsive to the micro-scale thermal gradient, and can they serve as practical indicators for diagnosing early-stage functional degradation? (3) Does the thermal stress drive coordinated shifts in trait covariance networks that reflect a systematic shift in ecological strategy? By explicitly treating AC exhaust as a microscale point-source compound stressor rather than as part of the conventional UHI background, this study addresses a neglected spatial scale of urban thermal heterogeneity and tests whether trait-based evidence can support more spatially precise vegetation monitoring, restoration, and planting design near anthropogenic heat sources.

## 2. Materials and Methods

### 2.1. Study Site and Experimental Design

The study was conducted within the urban area of Beijing, China (39°54′ N, 116°23′ E). Beijing has a typical warm-temperate semi-humid continental monsoon climate, with a mean annual temperature of 11–13 °C and mean annual precipitation of approximately 644 mm. January is the coldest month (mean −4 °C) and July the hottest (mean 26 °C), with summer maximum temperatures frequently reaching 35–40 °C. Field sampling and measurements were implemented in summer (June–August), the hottest and most humid season, which corresponds to the peak growing stage of urban vegetation.

Experimental plots were set in the Xibahe residential community, Chaoyang District, Beijing. All sampled trees were *F. chinensis* planted in 2020 from a common nursery source in Henan Province, according to the community greening management office. At the time of measurement, trees were seven years old, with a mean height of 3.7 m and mean diameter at breast height (DBH) of 8.9 cm. The community authority applied uniform management practices (irrigation, fertilization, and pruning) across all trees, ensuring relatively consistent soil and water conditions throughout the study area.

To minimize potential confounding effects, treatment and control trees were selected from the same residential community and along the same north-facing side of ground-floor residential buildings. Because the treatment and control trees were not arranged as one-to-one statistical pairs, the study was described as a matched treatment–control field comparison rather than a paired experimental design. Trees were chosen from comparable planting strips with similar surrounding hardscape conditions, building orientation, soil type, and site-management regime. The community authority applied uniform irrigation, fertilization, and pruning across all sampled trees, thereby reducing variation associated with maintenance practices and surface soil conditions. Treatment and control trees were further matched at the group level in terms of tree age (7 years), DBH range (8.5–9.3 cm), and canopy height range (3.4–4.0 m). These selection criteria were used to reduce the influence of non-AC-related factors such as shading, building proximity, reflected radiation, local soil heterogeneity, and rooting conditions, although such factors could not be completely eliminated in the field setting. Sixty trees located directly in front of continuously operating AC outdoor units at a distance of 2 m were selected as the treatment group, and another 60 trees free from AC exhaust influence and located more than 15 m from any heat source were assigned as the control group. The AC outdoor units were residential split-type models, with a rated cooling capacity of 3.5 kW, rated input power of approximately 1.0 kW, and outlet diameter of 0.55 m. All units were installed on the north-facing exterior walls of buildings, with the bottom edge approximately 0.5 m above ground level, and the outlets facing horizontally northward without obstructions. During the cooling season from June to August 2025, the units operated from 18:00 to 08:00, with exhaust air temperatures 5–15 °C above ambient levels. Occasional short interruptions of ≤10 min occurred due to temperature-regulation cycles, but the overall runtime exceeded 95%.

### 2.2. Microclimate Measurements

Thermo-hygrometers (HOBO MX2301A, Onset Computer Corporation, Bourne, MA, USA) were fixed at a height of 1.5 m to automatically record air temperature and relative humidity at 30 min intervals for over 30 consecutive days. Wind speed was measured three times each day with a portable anemometer (testo 410-1, Testo SE & Co. KGaA, Titisee-Neustadt, Germany). Because the AC outdoor units operated from 18:00 to 08:00, microclimate characterization was based on measurements collected during this operating period. Thus, the reported treatment–control differences in air temperature, relative humidity, and wind speed represent exposure-period differences during active AC exhaust, rather than full 24 h averages or conditions during the leaf-sampling window. For each day, all available records during 18:00–08:00 were averaged to obtain daily exposure-period means for subsequent statistical analysis.

### 2.3. Leaf Functional Trait Measurements

All leaf sampling activities were conducted between 10:00 and 10:30 a.m. Although sampling was performed after the nightly AC operating period, the measured leaf functional traits were interpreted as integrated responses to repeated exposure during the cooling season rather than as instantaneous responses to microclimatic conditions at the sampling moment. To ensure sampling consistency, leaves were collected from comparable middle-canopy positions, with canopy orientation, sampling height, and sampling time kept consistent across all trees. For each tree, ten intact, healthy, fully expanded, and mature leaves were randomly collected. All trait measurements were completed within 20 min after leaf detachment to minimize tissue water loss. Leaf functional traits were measured following standard protocols. Leaf thickness (LT) was measured at three equidistant positions on the leaf blade, excluding the midrib, using a digital micrometer with 0.001 cm precision, and the average value was recorded. Saturated fresh mass was determined after leaves were immersed in distilled water at 4 °C in the dark for 24 h, gently blotted dry, and immediately weighed. Leaf dry weight (LDW) was measured after oven-drying at 70 °C for 72 h to constant mass using an analytical balance with 0.001 g precision. For stomatal traits, nail-polish imprints were taken from the abaxial leaf surface and observed under 400× magnification. Three non-overlapping fields per leaf were analyzed using ImageJ to determine stomatal density (SD) and stomatal area (SA). For each tree, trait values from the ten sampled leaves were averaged to obtain tree-level trait values for statistical analyses; individual leaves were treated as technical subsamples rather than independent replicates. The calculation formulas, units, instruments, and measurement methods for the nine leaf functional traits are summarized in Table 1, with terminology and instrument formats standardized throughout the table.

### 2.4. Data Analysis

All statistical analyses were conducted in R 4.5.0 (R Foundation for Statistical Computing, Vienna, Austria). Figures were produced with the ggplot2 (version 3.5.2) package. Statistical significance was set at α = 0.05. Before analysis, we defined the experimental unit to avoid pseudoreplication. For microclimate data, repeated 30 min records were not treated as independent samples. We first calculated daily means for air temperature, relative humidity, and wind speed in each group. These daily means were then used for statistical comparisons (n = 32 days). For leaf traits, values from leaves collected from the same tree were averaged first. Each tree was then treated as one biological replicate. Thus, trait analyses used 60 independent trees per group. Individual leaves were treated as technical subsamples. Air temperature, relative humidity, and wind speed were compared between groups using Welch’s independent-samples *t*-tests. These tests were based on daily mean values. Results are reported as the mean ± standard deviation (SD). For the nine leaf traits, normality and variance homogeneity were checked using tree-level means. Independent-samples t-tests were used when assumptions were met. Mann–Whitney U tests were used when assumptions were not met. Effect sizes were calculated using Cohen’s d. Cohen’s d was recalculated from tree-level group means and pooled standard deviations using the formula d = (Mtreatment − Mcontrol)/SDpooled, where SDpooled = √[((ntreatment − 1)SDtreatment^2^ + (ncontrol − 1)SDcontrol^2^)/(ntreatment + ncontrol − 2)]. Signed d values were reported to show the direction of treatment effects, while absolute values |d| were used for effect-size classification. The effect size classes were defined as negligible (|d| < 0.2), small (0.2–0.5), medium (0.5–0.8), and large (>0.8). The log response ratio was also calculated as lnRR = ln(treatment mean/control mean). This value described the direction and size of trait responses [21]. Principal component analysis (PCA) was conducted using the nine standardized tree-level trait means. A biplot with 95% confidence ellipses was used to show group patterns in multivariate trait space. PERMANOVA was then used to test overall group differences in trait composition. This analysis used a Euclidean distance matrix and 999 permutations. A latent-variable structural equation model (SEM) was also built using LES theory. We used it as an exploratory path analysis. The aim was to examine hypothesized associations among AC-exhaust exposure, leaf economic strategy, and stomatal regulation. SEM was performed with the lavaan package in R 4.5.0. Treatment was coded as a binary variable. Control trees were coded as 0, and AC-exposed trees were coded as 1. Three endogenous latent variables were defined. Stress tolerance was indicated by LDMC, LTD, and LT. Resource acquisition was indicated by SLA, LA, CCI, and LDW. Stomatal regulation was indicated by SD and SA. Standardized path coefficients were reported to compare pathway strengths. Model fit was assessed using χ^2^, degrees of freedom, χ^2^/df, CFI, TLI, RMSEA with 90% confidence interval, SRMR, and AIC. R^2^ values were used only to describe explained variance in each endogenous latent variable. They were not used as measures of degradation severity or restoration potential. Direct, indirect, and total standardized associations were calculated from standardized path coefficients. These values were used for descriptive effect decomposition. Because this study used an observational matched treatment–control field comparison, SEM results were interpreted as hypothesized associations. They were not treated as definitive causal effects.

## 3. Results

### 3.1. Microclimate Characterization: Defining the Compound Stress Regime Associated with AC-Exhaust Exposure

Welch’s independent-samples *t*-tests based on daily exposure-period means confirmed that microclimatic conditions during the active AC operating period differed markedly between treatment and control sites (Table 2). The treatment sites showed higher air temperature, lower relative humidity, and higher wind speed than the control sites, indicating that trees located 2 m from operating AC outdoor units were repeatedly exposed to a distinct compound micro-environment characterized by elevated temperature, reduced humidity, and enhanced airflow. These microclimatic differences define the exposure background for interpreting subsequent leaf functional trait responses.

### 3.2. Univariate Trait Responses and Effect-Size Differentiation: Identifying Diagnostic Indicators of Functional Decline

**Figure 1:**
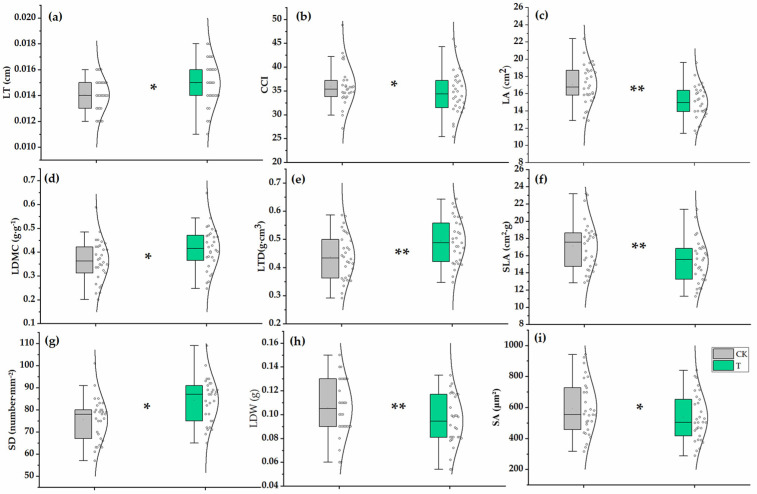
Responses of individual leaf functional traits. ((**a**) LT: Leaf thickness; (**b**) CCI: Chlorophyll content index; (**c**) LA: Leaf area; (**h**) LDW: Leaf dry weight; (**d**) LDMC: Leaf dry matter content; (**e**) LTD: Leaf tissue density; (**f**) SLA: Specific leaf area; (**g**) SD: Stomatal density; (**i**) SA: Stomatal area). Boxes represent the interquartile range, horizontal lines indicate medians, whiskers show data ranges, and points represent individual tree-level observations. Asterisks indicate significant differences between CK and T: * *p* < 0.05 and ** *p* < 0.01.

All nine leaf functional traits of *Fraxinus chinensis* trees growing 2 m from AC outdoor units differed significantly from those of the control group (all *p* < 0.05), with a highly consistent direction of variation (Figure 1). Collectively, leaves in the treatment group exhibited a more dense and conservative structural construction, whereas their resource-acquisition capacity and stomatal openness were significantly suppressed—patterns that signify a shift away from traits associated with high rates of transpirational cooling and carbon assimilation.

With respect to structural traits, the mean leaf thickness of the treatment group (0.0151 ± 0.0018 cm) was significantly greater than that of the control group (0.0142 ± 0.0014 cm; *p* = 0.028). Leaf dry matter content (LDMC) increased notably from 0.352 ± 0.090 g g^−1^ in the control group to 0.404 ± 0.096 g g^−1^ in the treatment group (+14.8%; *p* = 0.003). Leaf tissue density (LTD) was significantly higher in the treatment group (0.493 ± 0.081 g cm^−3^) than in the control group (0.434 ± 0.079 g cm^−3^; *p* = 0.006). For resource-acquisition traits, specific leaf area (SLA) in the treatment group (15.51 ± 2.84 cm^2^ g^−1^) was significantly lower than in the control group (17.35 ± 3.11 cm^2^ g^−1^; −10.6%; *p* = 0.012). Leaf area decreased from 16.98 ± 2.42 cm^2^ to 14.85 ± 2.21 cm^2^ (*p* = 0.007). Leaf dry weight declined from 0.105 ± 0.023 g to 0.093 ± 0.020 g (*p* = 0.015), and chlorophyll content index (CCI) was significantly lower in the treatment group (34.18 ± 4.74 vs. 35.81 ± 4.48; *p* = 0.041). Regarding stomatal traits, stomatal density in the treatment group (83.6 ± 10.1 no. mm^−2^) was 11.7% higher than in the control group (74.9 ± 9.2 no. mm^−2^; *p* = 0.008), whereas stomatal area was significantly reduced (553.5 ± 150.4 μm^2^ vs. 618.2 ± 166.4 μm^2^; *p* = 0.022). This “small-and-numerous” stomatal configuration, while adaptive for water conservation, implies a reduced capacity for transpirational cooling—a key regulating ecosystem service in urban settings.

After recalculation using tree-level means and pooled standard deviations, the signed Cohen’s d values showed that AC-exhaust exposure was associated with negative shifts in resource-acquisition traits and positive shifts in conservative or stomatal traits (Table 3). Based on the reported descriptive statistics, the largest standardized differences were observed for leaf area and stomatal density, followed by leaf tissue density, SLA, LDMC, leaf dry weight, and leaf thickness. CCI and stomatal area showed smaller standardized differences. Thus, leaf area and stomatal density showed the strongest statistical effect sizes, whereas LDMC, SLA, and stomatal density remain particularly practical candidate indicators because they are mechanistically linked to leaf economic strategy or stomatal configuration and can be measured relatively easily in field monitoring.

### 3.3. Multivariate Trait Space Separation: Evidence for Systematic Reconfiguration of Functional Strategy

**Figure 2:**
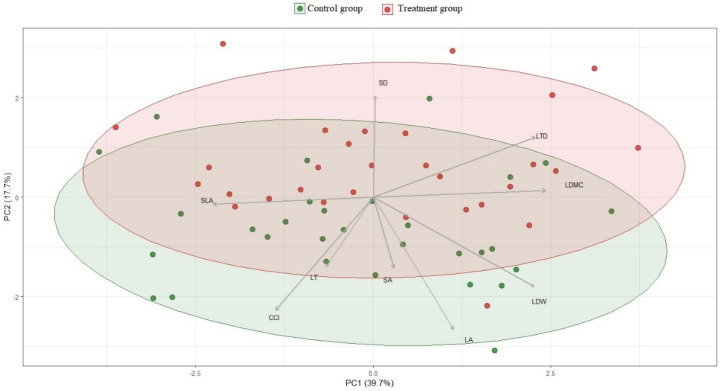
Principal component analysis (PCA) biplot of leaf functional traits for control and AC-exhaust treatment groups. Points represent individual tree-level observations, and ellipses indicate 95% confidence regions for each group. Arrows represent trait loading vectors. Axis labels indicate the variance explained by each component: PC1 = 39.7% and PC2 = 17.7%. PC1 reflects a leaf economics spectrum gradient from resource acquisition to conservative investment, whereas PC2 reflects variation in stomatal regulation and leaf morphological development. Trait abbreviations: LT, leaf thickness; CCI, chlorophyll content index; LA, leaf area; LDW, leaf dry weight; LDMC, leaf dry matter content; LTD, leaf tissue density; SLA, specific leaf area; SD, stomatal density; SA, stomatal area. The biplot shows a modest directional shift rather than complete separation between groups.

Principal component analysis (PCA) performed on the nine leaf functional traits yielded two principal components with eigenvalues > 1, together explaining 57.4% of total trait variance (PC1: 39.7%; PC2: 17.7%; Figure 2). PC1 was positively loaded by LDMC (0.486), LTD (0.457), and LDW (0.454), and negatively loaded by SLA (−0.450) and CCI (−0.274), representing the core leaf economic spectrum (LES) gradient from resource acquisition to conservative investment (Table 4). The positive end of PC1 reflected a more resource-conservative strategy. This pattern was linked to greater structural investment and lower resource acquisition. The negative end reflected a more resource-acquisitive strategy, with higher SLA and stronger acquisitive trait expression. The higher PC1 scores of treatment trees therefore suggest a directional shift toward a more conservative leaf economic strategy. This pattern indicates a partial group shift, not a complete separation between groups. PC2 was positively loaded by stomatal density (0.405). It was negatively loaded by leaf area (−0.531) and LDW (−0.360). This axis reflected variation in stomatal regulation and leaf morphological development. Treatment trees had higher PC2 scores. This pattern was consistent with higher stomatal density and smaller leaves. It suggests a shift toward a “small-and-numerous” stomatal configuration under AC-exhaust compound stress.

The treatment and control groups showed a partial separation in multivariate trait space. This separation was statistically significant, but it was not complete. The control group had a mean PC1 score of −0.22. The treatment group had a mean PC1 score of 0.22. This shift toward positive PC1 scores was linked to higher LDMC and LTD, and lower SLA. It suggests a modest shift toward the conservative end of the LES under AC-exhaust exposure. The treatment group also had a higher mean PC2 score than the control group (0.64 vs. −0.64). This pattern reflected higher stomatal density and smaller leaf area in treated trees. PERMANOVA showed a significant difference in nine-dimensional trait composition between groups (F = 5.89, R^2^ = 0.092, *p* = 0.001). Still, the low R^2^ value shows that treatment identity explained only 9.2% of total trait variation. Considerable variation also remained within each group. Thus, the PCA and PERMANOVA results indicate a modest but significant directional shift. They should not be read as strong or complete group separation. For monitoring, PCA-based trait combinations may help detect group-level functional shifts. These results should be interpreted together with sensitive single traits, such as LDMC, SLA, and stomatal density.

### 3.4. Exploratory Structural Equation Modeling: Hypothesized Associations Among AC-Exhaust Exposure, Leaf Economic Traits, and Stomatal Traits

**Figure 3:**
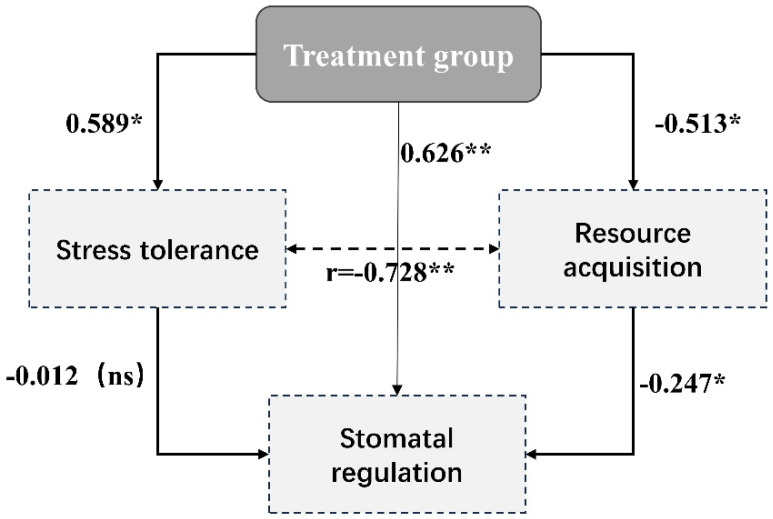
Exploratory structural equation model summarizing hypothesized associations among AC-exhaust exposure, leaf economic traits, and stomatal traits. Single-headed arrows represent standardized path coefficients (β), and the double-headed curved arrow denotes the correlation coefficient (r) between latent variables. Significance levels are indicated as follows: * *p* < 0.05, ** *p* < 0.01, and ns = not significant. Model fit indices were: χ^2^ = 23.6, df = 13, *p* = 0.04, χ^2^/df = 1.82, CFI = 0.96, TLI = 0.94, RMSEA = 0.06 (90% CI: 0.03–0.09), SRMR = 0.04, and AIC = 328.5.

Exploratory structural equation modeling was conducted with treatment group as the exogenous variable (0 = control, 1 = AC-exhaust treatment) and three latent variables—“Stress tolerance,” “Resource acquisition,” and “Stomatal regulation”—as endogenous variables (Figure 3; Table 5 and Table 6). Because this study was based on an observational matched treatment–control field comparison, the SEM results were interpreted as hypothesized associations rather than definitive causal effects.

AC-exhaust exposure was positively associated with stress tolerance (β = 0.589, *p* < 0.01) and negatively associated with resource acquisition (β = −0.513, *p* < 0.05). These associations suggest that exposed trees tended to show greater structural investment, as reflected by higher LDMC, LTD, and leaf thickness, while showing lower values of resource-acquisition traits, including SLA, leaf area, CCI, and leaf dry weight. Stress tolerance and resource acquisition were negatively correlated (r = −0.728, *p* < 0.001), consistent with the expected trade-off between conservative and acquisitive leaf economic strategies. AC-exhaust exposure was also associated with stomatal regulation (β = 0.626, *p* < 0.001). Resource acquisition was negatively associated with stomatal regulation (β = −0.247, *p* < 0.05), suggesting a potential indirect association linking lower resource-acquisition trait expression with altered stomatal traits. By contrast, the path from stress tolerance to stomatal regulation was not significant (β = −0.012, ns), indicating that structural conservatism was not significantly associated with stomatal regulation after accounting for the other pathways in the model.

To summarize the relative contributions of the modeled associations involving stomatal regulation, we conducted a descriptive decomposition of standardized path coefficients. The unmediated standardized association between AC-exhaust exposure and stomatal regulation was β = 0.626. The indirect association mediated by resource acquisition was calculated as the product of the two standardized path coefficients: (−0.513) × (−0.247) = 0.127. Therefore, the total modeled standardized association was 0.626 + 0.127 = 0.753. Within this exploratory model, the unmediated association accounted for 0.626/0.753 ≈ 83.1% of the total modeled association, whereas the indirect association through resource acquisition accounted for 0.127/0.753 ≈ 16.9%. Because the path from stress tolerance to stomatal regulation was not significant, it was not included in this descriptive decomposition. These values should be interpreted only as a summary of modeled associations, not as evidence of causal mechanisms or as a basis for quantitative restoration allocation.

The R^2^ values were interpreted only as the proportion of variance in each endogenous latent variable explained by the model predictors. The model explained 34.7% of the variance in stress tolerance, 26.4% of the variance in resource acquisition, and 59.8% of the variance in stomatal regulation (Table 6). These results indicate that, among the three endogenous latent variables, Stomatal regulation showed the highest proportion of explained variance in the exploratory model. Therefore, stomatal traits, particularly stomatal density, may serve as useful candidate indicators for monitoring AC-exhaust-associated leaf functional shifts. However, these SEM results should be regarded as association-based hypotheses that require further testing through controlled experiments, distance-gradient sampling, and direct physiological measurements.

## 4. Discussion

### 4.1. Micro-Scale Thermal Heterogeneity Associated with Functional Trait Shifts in Urban Trees

Our results provide field evidence that AC exhaust may function as a functionally significant “point-source heat landscape,” creating a discrete, high-contrast compound stress patch that generates steep environmental gradients over distances of only a few meters [13,22]. Importantly, this patch should not be interpreted as a simple temperature anomaly. Instead, AC exhaust simultaneously produced elevated air temperature, reduced relative humidity, and enhanced wind speed, thereby forming a compound heat–drought–wind micro-environment. Recent studies have shown that elevated vapor pressure deficit can reduce stomatal conductance, constrain carbon assimilation, and increase hydraulic risk, while long-term warming and elevated atmospheric demand may induce stomatal and leaf-trait acclimation. In addition, enhanced airflow can modify leaf boundary-layer conductance and increase gas exchange and transpiration demand. These findings support our interpretation that AC exhaust should be regarded as a compound heat–drought–wind stressor rather than a simple temperature anomaly [23,24,25]. Within this micro-scale patch, trees exhibited a coordinated shift toward the conservative end of the leaf economics spectrum (LES), evidenced by higher leaf dry matter content (LDMC), tissue density (LTD), and leaf thickness, coupled with lower specific leaf area (SLA), leaf area, and chlorophyll content (Table 2). The effect sizes observed (Cohen’s d = 0.39–0.59) are comparable to those reported along macro-scale urban–rural gradients [26,27], yet they emerged under single-season exposure to a highly localized compound point-source stress context. This suggests that point-source compound thermal–atmospheric heterogeneity may be associated with measurable functional trait shifts at the scale of individual trees, highlighting a fine-scale source of urban thermal heterogeneity that complements the traditional focus on city-wide urban heat island (UHI) effects.

The structural equation model summarized possible links among AC-exhaust exposure, leaf economic strategy, and stomatal regulation. AC-exhaust compound stress was positively associated with structural conservatism (β = 0.589). It was also negatively associated with resource acquisition capacity (β = −0.513) (Table 5). This pattern suggests a shift in exposed trees. They tended to move from a growth-oriented state toward a more conservative state. This shift may reduce some service-related functions. The strong negative correlation between the two dimensions was also important (r = −0.728). It suggests a possible trade-off between stress tolerance and resource acquisition [28,29]. Greater structural reinforcement may coincide with less photosynthetic area and lower cooling potential. Thus, the observed trait changes may reflect a coordinated shift in resource-use strategy. This shift was associated with AC-exhaust exposure. Still, the causal mechanisms need further testing. Controlled experiments and long-term monitoring are needed. This conservative shift is consistent with plant responses to heat and atmospheric drought. Similar patterns may occur in other northern-Chinese street trees. Examples include Platanus acerifolia and Sophora japonica. Both species have intermediate resource-use strategies. Yet species may differ in response strength. Evergreen species with conservative traits may show weaker shifts. Fast-growing species, such as Populus tomentosa, may be more sensitive under similar exposure. Because this study used one species, broad extrapolation remains limited. Future multi-species studies should compare different functional types. They should also identify general patterns and species-specific thresholds.

### 4.2. Spatial Footprint of Functional Degradation and Implications for Ecological Monitoring

A key question for urban green-infrastructure management is whether AC-exhaust exposure is associated with functional trait changes that extend beyond visually detectable structural symptoms. Our results suggest that such changes may occur at the leaf-trait level before obvious visual symptoms become apparent. The “small-and-numerous” stomatal configuration (density +11.7%, area −10.4%) observed in treated trees represents a regulatory adjustment that is not immediately visible, and may be linked to altered water-use regulation and gas-exchange potential [30]. However, because gas exchange, transpiration rate, and leaf-level cooling were not directly measured in this study, any implication for reduced transpirational cooling capacity should be interpreted as an indirect inference based on stomatal morphology rather than as direct physiological evidence. The SEM suggested that stomatal regulation was the functional dimension most strongly associated with the modeled predictors (R^2^ = 0.598). AC-exhaust exposure was directly associated with stomatal regulation (β = 0.626) and was also indirectly linked to this dimension through reduced resource-acquisition traits (β = −0.247). These results indicate a hypothesized association pathway linking AC-exhaust exposure, leaf economic traits, and stomatal traits, rather than confirming a “physiological degradation → regulatory degradation” amplification chain. Therefore, the functional footprint of a point-source heat landscape may extend beyond visible morphological change, but its consequences for ecosystem services such as transpiration-driven cooling require direct physiological measurements in future studies.

**Figure 4:**
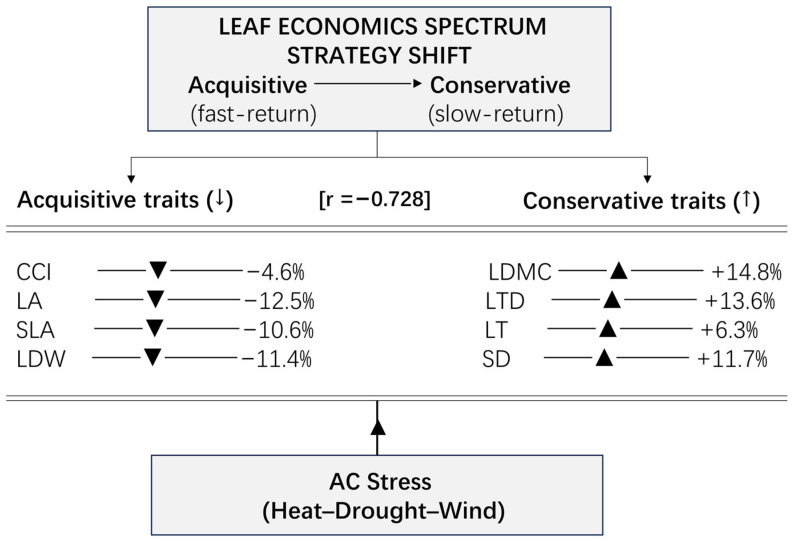
Leaf economics spectrum shift under AC exhaust stress. (LT: Leaf thickness; CCI: Chlorophyll content index; LA: Leaf area; LDW: Leaf dry weight; LDMC: Leaf dry matter content; LTD: Leaf tissue density; SLA: Specific leaf area; SD: Stomatal density). Arrows indicate the direction of trait change under AC-exhaust exposure. Downward arrows represent decreases in acquisitive traits, upward arrows represent increases in conservative traits, and the central upward arrow indicates AC-exhaust compound stress as the exposure background associated with the observed trait shifts.

These findings suggest a possible hierarchy of candidate monitoring traits (Figure 4). Stomatal density may be a useful early indicator. It responded clearly to AC-exhaust exposure. It can also be measured with epidermal imprints. LDMC and SLA may provide complementary information. They showed strong but opposite responses. They also require only simple gravimetric measurements. Including these traits in urban vegetation monitoring may help detect early leaf-level shifts. These shifts may appear before visible symptoms occur. Such symptoms include leaf chlorosis, curling, and premature senescence [17]. This approach could support earlier and more proactive management. SEM also helped summarize possible links among AC-exhaust exposure, leaf economic strategy, and stomatal regulation. Still, its results should be interpreted with caution. The matched treatment–control comparison can show associations between exposure and leaf traits. It cannot fully prove causality. It also cannot exclude all remaining site-level differences. The pathways identified here should therefore be treated as plausible ecological interpretations. They need further testing. Future studies should use controlled experiments or long-term monitoring. They should test temperature, humidity, airflow, soil water, and exposure duration separately. They should also measure gas exchange and cooling directly. This would show whether the observed stomatal and LES trait shifts lead to measurable changes in transpiration-driven cooling services [31,32].

### 4.3. From Trait Associations to Cautious Management Hypotheses

The quantitative trait-response patterns and exploratory SEM results reported in this study provide a preliminary basis for identifying candidate monitoring indicators and for developing cautious management hypotheses near AC outdoor units. The observed changes in LDMC, SLA, leaf area, stomatal density, and stomatal area suggest that trees located 2 m from operating AC units may experience measurable shifts in leaf economic and stomatal traits. These shifts were associated with a localized compound heat–drought–wind micro-environment generated by AC exhaust. However, because this study was based on an observational matched treatment–control comparison, these results should be interpreted as field-based associations rather than definitive causal evidence or direct restoration prescriptions.

The exploratory SEM provided a useful summary of hypothesized associations among AC-exhaust exposure, resource-acquisition traits, stress-tolerance traits, and stomatal traits. The standardized association between AC-exhaust exposure and stomatal regulation was stronger than the indirect association mediated through resource acquisition. This pattern suggests that reducing direct exposure to AC-exhaust plumes may be a reasonable precautionary management option. Possible measures include avoiding direct plume contact with tree crowns, redirecting exhaust airflow away from vegetation, installing physical barriers where appropriate, or improving local maintenance practices such as irrigation and soil management. Nevertheless, these measures should be regarded as management hypotheses requiring further validation rather than confirmed design guidelines.

Importantly, the present study cannot define fixed planting-distance thresholds. Our sampling design compared trees located 2 m from AC outdoor units with control trees located more than 15 m from heat sources, but it did not include trees at intermediate distances such as 3 m, 4 m, or other points along a continuous distance gradient. Therefore, the data do not support a minimum planting distance of 4 m, nor do they support a fixed planting-zone framework. Any distance-related recommendation would require future studies that explicitly sample along distance gradients and evaluate how microclimate intensity and leaf functional traits change with increasing distance from AC exhaust outlets.

Future research should combine distance-gradient sampling, controlled experiments, and direct physiological measurements. Distance-gradient studies should include multiple distances from AC outlets, such as 1 m, 2 m, 3 m, 4 m, and greater distances, to test whether leaf trait responses decline gradually or show threshold-like patterns. Controlled experiments should separately manipulate temperature, humidity, airflow, soil water availability, and exposure duration to distinguish the relative contribution of each stress component. Direct measurements of stomatal conductance, photosynthetic rate, transpiration, leaf temperature, and whole-tree cooling performance are also needed to determine whether the observed trait shifts translate into measurable changes in physiological function or ecosystem-service provision.

More broadly, the present results suggest that leaf functional traits may be useful for monitoring fine-scale anthropogenic stress in urban green infrastructure. Traits such as LDMC, SLA, and stomatal density are relatively practical to measure and may help detect early leaf-level responses before obvious visual symptoms appear. These indicators could be combined with high-resolution microclimate monitoring and thermal mapping to identify potential exposure hotspots near anthropogenic heat sources. However, such monitoring should be used to guide adaptive management and future hypothesis testing, not to establish mandatory planting-distance standards at this stage.

## 5. Conclusions

This study showed that repeated exposure to an AC-exhaust-associated compound heat–drought–wind micro-environment was associated with coordinated shifts in leaf functional traits of *Fraxinus chinensis* street trees. Compared with control trees located more than 15 m from heat sources, trees located 2 m from operating AC outdoor units showed higher leaf dry matter content, leaf tissue density, leaf thickness, and stomatal density, together with lower specific leaf area, leaf area, leaf dry weight, chlorophyll content index, and stomatal area. These patterns suggest a shift toward more resource-conservative leaf trait expression under localized AC-exhaust exposure.

Leaf dry matter content, specific leaf area, and stomatal density emerged as practical candidate indicators for monitoring AC-exhaust-associated leaf functional shifts. These traits are relatively easy to measure and are closely related to leaf economic strategy and stomatal configuration. Therefore, they may be useful for detecting early leaf-level responses to fine-scale anthropogenic thermal stress before obvious visual symptoms become apparent. However, their value as diagnostic indicators should be further tested across different tree species, exposure durations, and urban micro-environmental settings. Exploratory structural equation modeling summarized hypothesized associations among AC-exhaust exposure, leaf economic traits, and stomatal traits. The model results should be interpreted as association-based hypotheses rather than definitive causal mechanisms, because this study was based on an observational matched treatment–control field comparison. The R^2^ values should be understood only as the proportion of variance explained by the model predictors, not as evidence of impairment severity, degradation intensity, or restoration potential.

These findings support precautionary management near active AC outdoor units, such as reducing direct plume contact with tree crowns, redirecting exhaust airflow away from vegetation, installing physical barriers where appropriate, and improving local maintenance practices. However, because this study sampled trees only at 2 m from AC units and compared them with controls located more than 15 m from heat sources, it cannot define fixed planting-distance thresholds or evidence-based zoning boundaries. In particular, the present data do not support a 4 m minimum planting distance or a fixed three-tier planting-zone framework.

Future studies should use distance-gradient sampling and manipulative experiments to test whether leaf trait responses persist, weaken, or disappear at 3 m, 4 m, or greater distances from AC exhaust outlets. Further work should also directly measure stomatal conductance, photosynthetic rate, transpiration, leaf temperature, tree growth, and cooling performance to determine whether the observed trait shifts translate into measurable physiological changes or altered ecosystem-service provision. Multi-species studies are also needed to evaluate species-specific sensitivity and to develop more reliable, evidence-based guidance for urban planting and adaptive management near anthropogenic point-source heat emissions.

## Figures and Tables

**Table 1:** Leaf functional traits measured in this study.

Trait Category	Trait	Abbreviation	Unit	Method/Instrument
Structural trait	Leaf thickness	LT	cm	Digital micrometer
Biochemical trait	Chlorophyll content index	CCI	–	CCM-200 chlorophyll meter (Opti-Sciences, Inc., Hudson, NH, USA)
Morphological trait	Leaf area	LA	cm^2^	Flatbed scanner combined with ImageJ software (version 2.15.0; National Institutes of Health, Bethesda, MD, USA)
Biomass trait	Leaf dry weight	LDW	g	Dried in an oven at 70 °C until constant mass
Economics spectrum trait	Leaf dry matter content	LDMC	g g^−1^	LDMC = leaf dry mass/saturated fresh mass
Economics spectrum trait	Leaf tissue density	LTD	g cm^−3^	LTD = leaf dry mass/(leaf area × leaf thickness)
Economics spectrum trait	Specific leaf area	SLA	cm^2^ g^−1^	SLA = leaf area/leaf dry mass
Stomatal trait	Stomatal density	SD	no. mm^−2^	Nail-polish imprint method combined with light microscopy
Stomatal trait	Stomatal area	SA	μm^2^	Light microscopy observation and ImageJ analysis

**Table 2:** Descriptive statistics and Welch’s independent-samples *t*-test results for microclimate variables during the active AC operating period from 18:00 to 08:00.

Variable	Group	n Days	Mean ± SD	Range	t-Value	*p*-Value
Air temperature (°C)	Treatment	30	38.3 ± 0.38	37.5–38.9	74.9	<0.001
	Control	30	32.0 ± 0.26	31.6–32.5		
Relative humidity (%)	Treatment	30	42.5 ± 0.78	41.4–44.2	−72.3	<0.001
	Control	30	54.8 ± 0.51	53.8–55.6		
Wind speed (m s^−1^)	Treatment	30	1.7 ± 0.2	1.4–2.1	32.9	<0.001
	Control	30	0.33 ± 0.11	0.2–0.6		

Note: The large t-values reflect the consistently large treatment–control differences relative to the low day-to-day variability in daily exposure-period means. They do not result from treating repeated 30 min measurements as independent observations.

**Table 3:** Effect-size classification of leaf functional traits based on Cohen’s d.

Trait	Direction of Response	Approximate Signed Cohen’s d	Magnitude
LT	Increase	0.56	Medium
CCI	Decrease	−0.35	Small
LA	Decrease	−0.92	Large
LDW	Decrease	−0.56	Medium
LDMC	Increase	0.56	Medium
LTD	Increase	0.74	Medium
SLA	Decrease	−0.62	Medium
SD	Increase	0.9	Large
SA	Decrease	−0.41	Small

Note: Cohen’s d values were calculated using tree-level means and pooled standard deviations. Signed values indicate the direction of the treatment effect relative to the control group; absolute values were used for magnitude classification. Values shown in this table were recalculated from the raw tree-level data. LT, leaf thickness; CCI, chlorophyll content index; LA, leaf area; LDW, leaf dry weight; LDMC, leaf dry matter content; LTD, leaf tissue density; SLA, specific leaf area; SD, stomatal density; SA, stomatal area.

**Table 4:** Load of leaf functional traits on the first two principal components.

Trait	LT	CCI	LA	LDW	LDMC	LTD	SLA	SD	SA
PC1	−0.133	−0.274	0.226	0.454	0.486	0.457	−0.450	0.006	0.058
PC2	−0.279	−0.453	−0.531	−0.360	0.027	0.241	−0.028	0.405	−0.285

**Table 5:** Standardized path coefficients from the exploratory structural equation model. Coefficients represent hypothesized associations rather than confirmed causal effects.

Functional Degradation Pathway	Standardized Coefficient (β)	*p*-Value	Interpretation of Degradation Mechanism
Direct effects			
Heat stress → Stress tolerance (Structural degradation)	0.589	**	Heat stress directly induces leaf structural conservatism
Heat stress → Resource acquisition (Physiological degradation)	−0.513	*	Heat stress directly suppresses photosynthetic and growth functions
Heat stress → Stomatal regulation (Regulatory degradation)	0.626	***	Heat stress directly drives abnormal stomatal functional adjustment
Stress tolerance → Stomatal regulation	−0.012	ns	Structural degradation is not directly transmitted to stomatal regulation
Resource acquisition → Stomatal regulation	−0.247	*	Cascading pathway from physiological function degradation to regulatory function degradation
Covariance (correlation)			
Stress tolerance ↔ Resource acquisition	−0.728	***	Core trade-off between structural defense and physiological gain: cannot be simultaneously optimized in restoration design

Note: ns = not significant; * *p* < 0.05; ** *p* < 0.01; *** *p* < 0.001.

**Table 6:** Coefficients of determination (R^2^) for endogenous latent variables in the exploratory structural equation model. R^2^ values indicate the proportion of variance explained by the model predictors.

Endogenous Latent Variable (Functional Degradation Dimension)	R^2^	Interpretation of Variance Explained by Model Predictors
Stress tolerance (Structural degradation)	0.347	The model explained 34.7% of the variance in stress-tolerance traits.
Resource acquisition (Physiological degradation)	0.264	The model explained 26.4% of the variance in resource-acquisition traits.
Stomatal regulation (Regulatory degradation)	0.598	The model explained 59.8% of the variance in stomatal regulation traits, the highest among the three endogenous latent variables.

## Data Availability

The data presented in this study are available on request from the corresponding author. The data are not publicly available due to institutional policies and because the original field records are part of an ongoing research project.
